# A Nexus of Biomolecular Complexities in Pituitary Neuroendocrine Tumors: Insights into Key Molecular Drivers

**DOI:** 10.3390/biomedicines13040968

**Published:** 2025-04-16

**Authors:** Ligia Gabriela Tataranu

**Affiliations:** 1 Department of Neurosurgery, Carol Davila University of Medicine and Pharmacy, 020021 Bucharest, Romania; ligia.tataranu@umfcd.ro; 2 Department of Neurosurgery, Bagdasar-Arseni Emergency Clinical Hospital, 041915 Bucharest, Romania

**Keywords:** pituitary neuroendocrine tumors, tumor microenvironment, pituitary, molecular pathology, cellular and molecular biology

## Abstract

Approximately 90% of the lesions of hypophyseal origins are represented by pituitary neuroendocrine tumors, which further account for up to 22.5% of the intracranial tumors in the adult population. Although the intricacy of this pathology is yet to be fully understood on a biomolecular level, it is well known that these lesions develop within a microenvironment that supports their evolution and existence. The role of the tumoral microenvironment in pituitary lesions is pivotal, mainly due to this gland’s distinct anatomical, histological, and physiological structure and function. Each component of the tumoral microenvironment is specifically involved in tumorigenesis, angiogenesis, tumoral growth, progression, and dissemination. By recognizing and understanding how these elements are involved in such processes, targeted treatments can emerge, and better future management of pituitary lesions can be provided. This article aims to summarize the role of each component of the tumoral microenvironment in pituitary lesions while assessing their association with biomolecular mechanisms.

## 1. Introduction

The cellular environment in which a tumor develops and exists comprises an extracellular matrix, new vascular networks, signaling molecules, tumoral cells, and non-tumoral cells such as fibroblasts or migrated immune cells [1]. This environment has been referred to as a tumoral microenvironment (TME). Pivotal factors like hypoxia, acidity, or growth factors will majorly impact tumoral development. Regarding its origin and heterogeneity, it is important to mention that a harmonious orchestration of changes is involved. The development of a tumor is initiated by the expansion of neoplastic cells. Following this process, all of the elements mentioned earlier that comprise the TME will simultaneously evolve with the neoplastic cells and actively participate in the mechanism of tumorigenesis [1]. Furthermore, the TME not only supports neoplastic processes but also promotes them, and the difference between a normal cellular environment and the TME is significant [2]. For example, some components of the TME have a different gene expression profile than normal healthy components [3]. The tumor-associated stroma will have a different biomolecular structure and, unlike the normal stroma, will support tumoral growth. Tumor-associated fibroblasts will also support tumoral growth and metastasis, and in comparison to normal fibroblasts, they will exhibit alterations in glucocorticoid receptor-induced gene transcription [3]. Although these are just a few examples, we will discuss all the components of the TME and their implications in pituitary lesions.

Besides the significant role in tumoral growth and support, the TME is also involved in therapeutic management and drug resistance, as the tumor niche shapes the treatment response [4,5,6,7]. Multi-targeted agents have been proven beneficial when addressing various TME compartments [8,9]. Thus, preclinical and clinical data demonstrated promising results with drugs in therapeutic combinations or single drugs, targeting stromal fibroblasts, vasculature, and immune cells [1,8,10]. Moreover, in monotherapy or combinations, different strategies regarding targeted therapeutic approaches were successfully implemented with agents such as Ipilimumab, Nivolumab, and Pembrolizumab [11,12], while others like mammalian target of rapamycin (mTOR), tyrosine kinase, and vascular endothelial growth factor (VEGF) inhibitors are still under investigation [13]. This is the case for targeting the hypoxic milieu, blocking the biomolecular dialog between TME components and tumoral cells, and interfering with abnormal intracellular signaling pathways [14].

From a general perspective, the TME has a pivotal role in tumor formation and growth, as well as treatment resistance, and disrupting the biomolecular mechanisms in this environment can offer a superior level of therapeutic intervention [14].

**Figure 1:**
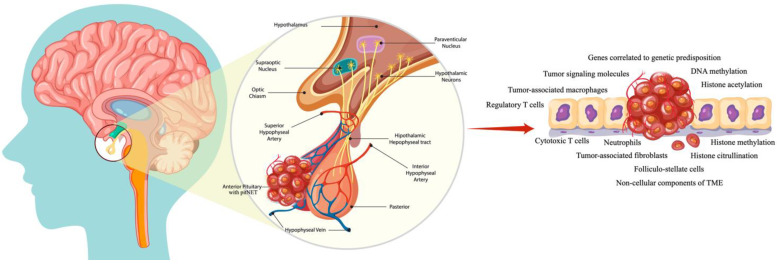
A visual representation of the tumor microenvironment’s involvement in pituitary lesions. The PitNETs arise from the anterior hypophysis; thus, tumoral masses developing here can not only impact hormonal production but also disturb the homeostasis of the entire gland. Consequently, all tumor microenvironment components will have a different impact on supporting the survival of formed lesions and further development and/or aggressiveness.

To conclude, it is worth mentioning that in recent decades, major advancements have been made regarding the TME. Albeit still on the surface of knowledge, preclinical and clinical studies have demonstrated a deeper understanding of this matter [15]. A visual summary of the current article is represented in Figure 1.

## 2. Biomolecular Drivers

The most frequently encountered pituitary lesions are represented by pituitary adenomas, recently named pituitary neuroendocrine tumors (PitNETs). These tumors comprise approximately 90% of pituitary lesions, which further account for up to 22.5% of the intracranial tumors in the adult population [16]. Given that the pituitary gland is responsible for general homeostasis, it is understandable that any dysfunction can cause morbidity and mortality [17]. Pituitary lesions develop within a microenvironment that supports their evolution and existence. The concept of the TME in pituitary tumors is intricate and yet to be fully understood; however, it is well known to favor overall survival and growth. The TME contributes to tumorigenesis not only by being involved in genetic and epigenetic mechanisms but also by providing biochemical and physical cues that will interfere with cellular behaviors [18]. Furthermore, the TME is also a result of the crosstalk between neighboring immune cells and neoplastic cells and is a continuously evolving entity [19].

Notwithstanding the initially proposed hypothesis of a monoclonal origin of pituitary tumors, various research studies identified multiple stem/progenitor cells in these lesions [20]. Although most pituitary lesions are sporadic, in approximately 5% of the cases, they can develop as part of a genetic syndrome or predisposition. In cases of genetic syndromes, specific genes have been discovered to be linked to their pathogenesis, and in very rare cases, these genes are also involved in the pathogenesis of sporadic tumors [21].

### 2.1. A Brief Overview of Tumor-Signaling Molecules and Genes Correlated with Genetic Predisposition in PitNETs

The modern scientific approach to tumorigenesis regards transforming healthy cells into neoplastic ones and highlights the importance of the TME. In PitNETs, this microenvironment comprises host cells, secreted factors, the extracellular matrix, tumor signaling molecules, and genes correlated to genetic predisposition [22]. To initiate this discussion, we will consider a brief overview of the biomolecular aspects of tumor-signaling molecules and genes correlated with genetic predisposition in PitNETs.

*Multiple endocrine neoplasia type 1 (MEN1)* has a tumor suppressor function and is involved in cellular proliferation, gene transcription, and genome stability. The tumor types associated with *MEN1* are represented by lactotroph, somatotroph, corticotroph, and nonsecreting adenomas [23].

*Cyclin-dependent kinase inhibitor 1B (CDKN1B)* is a gene located on 12p13.1. Although it is susceptible to somatotrophinomas development, it can also be involved in other PitNET types, and it is involved in cell cycle regulation [24].

Located on 17q24.2, *protein kinase cAMP-dependent type I regulatory subunit alpha (PRKAR1A)* is also related to the tumorigenesis of somatotroph and lactotroph adenomas, and it has been stated that its loss enhances protein kinase A (PKA) signaling [24].

An alteration of the *G-protein-coupled receptor (GPR101)* may lead to the activation of the cAMP-PKA pathway, and it is associated with the development of somatotroph adenomas [24]. It has been hypothesized that by modulating *VEGF* expression and influencing apoptosis, the *Von Hippel–Lindau (VHL)* gene is involved in tumorigenesis [25].

*Dicer 1*, *ribonuclease III (DICER1)* is associated with pituitary blastoma, while *MutL homolog 1 (MLH1)* and *MutS homolog 2 (MSH2)* are associated with corticotroph adenomas [23,26]. The *aryl hydrocarbon receptor-interacting protein (AIP)* interacts with the synthesis of cAMP, and it has been found to be connected to all PitNET types [27].

Located on 20q13.32, the *Guanine nucleotide-activating subunit (GNAS)* is mostly involved in somatotroph adenomas, acting upon cAMP levels and PKA when activated, while *succinate dehydrogenase x (SDHx)* is also a gene that has been linked to PitNETs, although its function is still in research [24,28]. However, recent studies concluded that germline mutations in *SDHx* were demonstrated in hereditary pituitary lesions, such as phaeochromocytoma/paraganglioma, with pituitary adenoma [24].

*Pituitary tumor-transforming gene-1 (PTTG1)* is located on 5q33.3 and, by driving chromosomal instability, leads to the tumorigenesis of all types of PitNETs [29,30]. *Signal transducer and activator of transcription 3 (STAT3)* is located on 17q21.2, and its enhancement increases GH transcription, supporting the development of somatotroph adenomas [31,32]. *Cadherin-related 23 (CDH23)* is also associated most frequently with somatotroph adenomas [33]. Mutations in *immunoglobulin superfamily member 1 (IGSF1)* increase GH secretion and IGF-1 levels, and it is associated with pituitary hyperplasia [34]. *PR domain zinc finger protein (PRDM2)* has a function in *cellular myelocytomatosis oncogene (c-Myc)* regulation, while increased expression of *solute carrier family 20 member 1 (SLC20A1)* has been linked to the activation of the Wnt–b-catenin signaling pathway [35,36].

The lack of the *PR/SET Domain 2* gene has been incriminated in PitNET development through the regulation of c-Myc. *PRDM2*, *SLC20A1*, *SSTR1-5*, and *PR/SET Domain 2* are observed to be involved in the development of somatotroph adenomas [35,36,37]. Decreased expression of *somatostatin receptors/(SSTR1-5)* and dopamine receptors *(SSTRs and DRDs*), such as *DRD4*, *DRD5*, *SSTR1*, and *SSTR2*, has been associated with the tumorigenesis of somatotroph adenomas [38].

The *growth arrest and DNA-damage-inducible gamma (GADD45γ)* tumor suppressor gene may be involved in the development of somatotroph and nonfunctioning adenomas through multiple mechanisms. One of them is DNA damage and function in the negative regulation of cell growth [38].

While its mechanisms are still unknown, in all types of PitNETs, promoter methylation of *ras association domain family member 1 (RASSF1A)* has been observed [39].

*Galectin 3 (LGALS3)* has been incriminated, as well, in the development of lactotroph and corticotroph adenomas [40]. *B-Raf proto-oncogene*, *serine/threonine kinase (BRAF)* activates MAPK and increases *proopiomelanocortin gene (POMC)* expression, while the overexpression of *secreted frizzled-related protein 2 (SFRP2)* reduces b-catenin and decreases Wnt signaling activity [41,42].

*Fibroblast growth factor receptor 2 (FGFR2)* has a mechanism responsible for inducing Rb phosphorylation and the regulation of cell cycle progression via p21 and p27. This is primarily performed through deubiquitination. Thus, *ubiquitin-specific peptidase 8 (USP8)* and *ubiquitin-specific peptidase 48 (USP48)* have been linked to the pathogenesis of PitNETs. *USP8*, *USP48*, *BRAF*, *SFRP2*, and *FGFR2* are related to the development of corticotroph adenomas [39,42].

Through unknown mechanisms, *heat shock protein 90 (USP90)*, *histone deacetylase 2 (HDAC2)*, *Cdk5 and Abl enzyme substrate 1 (CABLES1)*, the *pituitary tumor apoptosis gene (PTAG)*, *thrombospondin-1 (TSP-1)*, and *caspase-8 (CASP-8)* are also involved in the tumorigenesis of corticotrophinomas [23].

*C5orf66 antisense RNA 1 (C5orf66-AS1)* and *ectodermal-neural cortex 1 (ENC1)* also have unknown mechanisms of working, but it is well known that these biomarkers are involved in the development of null cell adenomas [43,44]. Also, through unknown mechanisms, *family with sequence similarity 90 member a1 (FAM90A1)*, *inhibitor of growth family member 2 (ING2)*, *ETS proto-oncogene 2*, *transcription factor (ETS2)*, *signal transducer and activator of transcription 6 (STAT6)*, *myelin transcription factor 1 like (MYT1L)*, and *potassium two pore domain channel subfamily k member 1 (KCNK1)* are involved in tumoral regrowth [45].

In addition, in nonfunctioning adenomas, *interleukin 6 receptor (IL-6R)/Janus kinase 2 (JAK2)/STAT3/matrix metallopeptidase 9 (MMP9)* and *phosphatidylinositol 3-kinases (PI3K)* have been discovered to be involved in the PI3K/AKT pathway. This correlates with cell survival, growth, proliferation, and metabolism [46]. *Maternally expressed 3 (MEG3)* acts as a tumor genesis suppressor through both p53-dependent and p53-independent pathways. Furthermore, *cyclin-dependent kinase inhibitor 2A (CDKN2A)* is a tumor suppressor and regulates the cell cycle; however, both genes are featured in the tumorigenesis of nonfunctioning adenomas and somatotroph adenomas [23,47,48]. Table 1 summarizes the most important genes involved in PitNETs and their main roles.

### 2.2. The Landscape of Molecular Events in Pituitary Tumor Apoplexy

Pituitary tumor apoplexy (PA) is defined by intratumoral hemorrhage and/or infarction. An early diagnosis may be of crucial significance, as it can be a life-threatening condition, and prompt medical and surgical treatment may be essential [51]. Even though, in this case, the molecular pathways in their entirety are still not clarified, it is well known that a small vascularization of PitNETs is consequential, as it triggers various biomolecular mediators responsible for tumoral hemorrhage [52].

Various genetic factors are involved in PA, each of which has a specific role. VEGF is mainly involved in tumor angiogenesis; endolin (CD105, CD31) has a role in microvascular density, while the pituitary tumor-transforming gene and fibroblast growth factor are involved in pituitary tumorigenesis and development. In contrast, the Ki67 marker has a role in cellular proliferation [53]. Furthermore, it is worth mentioning the involvement of tumor necrosis factor alpha in angiogenesis, vascular hyperpermeability, and the destruction of vascular integrity; the role of hypoxia-inducible factor 1-alpha (HIF-1α) in hypoxia, and the activation of VEGF; and the role of matrix metalloproteinase-2/9 (MMP 2/9) in the degradation of the extracellular matrix and vascular permeability [53].

In PitNETs, HIF-1α increases the risk of PA by stimulating the expression and synthesis of VEGF (which influences MAPK, FAK, PI3K/Akt, and p38 MAP kinase signaling pathways), MMP 2/9, and TGF-β, as well as the Wnt signaling pathway. PA is more common in non-functioning macroadenomas (showing elevated microRNA values for VEGF) than in pituitary microadenomas [53,54,55]. However, further research is needed to gain more knowledge about the biomolecular pathways leading to pituitary apoplexy, which will improve the management of this condition.

### 2.3. Non-Invasive Circulating Biomarkers in PitNETs

PitNETs are habitually managed through a neurosurgical transsphenoidal approach, and a solid tissue biopsy is used to diagnose the tumor. However, this technique may offer a single-time sample, and the genetic heterogeneity of the tumor is not always captured. Finally, this may lead to the loss of significant genetic information. Contrariwise, a liquid biopsy is a procedure used to detect the biomolecular elements of the tumors in the biofluids, such as blood, cerebrospinal fluid, urine, and saliva, even though the urine is the least explored and the blood is the most investigated one [29]. This technique is accessible and minimally invasive, providing an early screening of the tumoral mass and a prompt diagnosis. Multiple collected samples may provide a thorough insight into the genetic heterogeneity and tumoral evolution of PitNETs [56].

Similar to solid tissue sampling, biofluids carry all clinical necessary parameters for analysis (proteins, DNA, RNA) and have widely been used in the diagnosis, prognosis, and management of multiple diseases, including PitNETs [57].

Although small tumors may release low titers of biomarkers, the vascularization of the pituitary gland may offer a significant connection between the circulation and its cells [58]. Recent studies reveal that, on average, tumors may have up to 80 different mutations and many more methylation transpositions [59].

Several typical mutations identified in pituitary lesions, such as in *GNAS* or *USP8*, *USP48*, *HHIPL1*, *NNAT*, *RHOU*, *C5orf66*, and *RASSF3*, have not yet been described in the biofluids. A notable fact is that better specificity has been observed in methylation profiles as they are more recurrent [29]. In summary, specific PitNET-related biomarkers in the liquid biopsy provide crucial details about tumoral behavior and origin. These are mainly represented by the circulating tumor DNA, cell-free RNA, epigenetic factors, and circulating tumor cells [29].

#### 2.3.1. Circulating Tumor DNA (ctDNA)

The circulating DNA is represented by fragments of DNA liberated into the blood circulation, making it preserve all its initial genetic and epigenetic features, and it can be a promising tool for liquid biopsy in PitNETs [60]. Benign tumors are supposed to release lower amounts of ctDNA through extracellular vesicles that protect their cargo from plasma nucleases (exosomes) [61]. Repeated sampling can provide details regarding tumoral progression, consistently bringing forth the latest information about the tumor [62].

#### 2.3.2. Cell-Free RNA (Long Non-Coding RNAs, Messenger RNA, and Micro-RNAs)

A specific type of RNA found in biofluids is represented by the cell-free RNA (cf-RNA) that acts on the DNA or proteins, unlike messenger RNA (mRNA), which seldom codes for the last. Until now, PitNETs are correlated with the long non-coding RNA (lncRNA), messenger RNA (mRNA), and micro-RNA (miRNA) [29].

LncRNA has been detected in all adenoma subtypes; miRNA has been detected in gonadotropinomas and somatotropinomas, and mRNA has been detected in non-functioning PitNETs [63,64,65,66]. To date, the only studied lncRNA in biofluids in association with PitNETs is the oncogene H19. However, many others are still available for further research, such as circular RNA, small nucleolar RNA, and piwi-interacting RNA. These biomolecular agents are highly stable in biofluids and are of great interest as they are associated with tumor development and progression [67,68,69].

#### 2.3.3. Epigenetic Factors

The term epigenetic refers to an alteration in gene expression without changes in the DNA sequence, and these alterations have a pivotal role in tumoral development [70].

The use of methylation markers in liquid biopsy for prognosis in PitNETs is not very common, and further research is still required. However, it is worth mentioning that the overexpression of DNA methyltransferases 1 and 3 has been reported in macroadenomas. These findings suggest that the inhibition of these enzymes will lead to an antitumoral effect [71].

Other described epigenetic changes in PitNETs are represented by DNA methylation, histone acetylation, histone methylation, and histone citrullination. The genes involved in cellular growth and signaling that exhibit altered methylation status are as follows: *CDK1*, *CDKN1B*, *CDKN2A*, *CDKN2C*, *retinoblastoma transcriptional corepressor 1 (RB1)*, *CDKN2A protein (p16INK4a)*, *retinoblastoma (Rb)*, the *CDKN1B protein (p27kip1)*, *GADD45γ*, *RASSF1A*, *RASSF3*, *apoptotic regulators*, the *pituitary tumor apoptosis gene (PTAG)*, *MEG3*, and *FGFR2* [70].

Regarding histone modifications, it has been stated that non-invasive PitNETs express higher amounts of the anti-PRDM2 antibody (RIZ1), acting as tumor suppressors and as histone methyltransferase in comparison to invasive PitNETs [72]. Furthermore, the overexpression of RIZ1 has been associated with important differences in methylation in multiple CpG sites, reduced H3K4/H3K9 methylation, enhanced H3K27 methylation, and longer survival rates [72]. In conclusion, certain associations between epigenetic changes and genetic alterations support the possibility that histone changes may have an impact on gene expression in PitNETs [70].

#### 2.3.4. Circulating Tumor Cells

Although they are very rare in benign tumors, circulating tumor cells (CTCs), which represent parts shredded from the original tumor into the bloodstream, can be difficult to isolate due to their biomolecular characteristics and low titer [73]. However, it has been demonstrated that they can be detected in PitNETs and not only in malignant tumors, which may suggest that pituitary carcinomas arise from adenomas [73]. Even though their beneficial part is still not clear, research regarding the matter is still in progress [74].

## 3. Tumoral Microenvironment

### 3.1. Immune and Stromal Cells

In addition to the biomolecular aspects regarding signaling pathways and genetic predispositions, the TME is also a result of interactions between its components and a highly advanced physical structure that supports tumoral development, growth, and survival [22]. The intricacy of immune and stromal cells within the TME in PitNETs has been highlighted in recent decades, while understanding that they can either have pro- or anti-tumorigenic functions based on their type [75].

#### 3.1.1. Macrophages

As the most abundant immune population in the TME, accounting for up to 50% of solid tumoral lesions, tumor-associated macrophages represent a heterogeneous cell population, providing trophic and nutritional support for neoplastic cells. This support will eventually lead to the progression of the disease and, most importantly, to treatment resistance. This is one of the main reasons why they are such an important entity that sparks significant interest in scientific oncological and biomolecular research [76]. It is worth mentioning that, as in other tumoral types, an increased level of macrophage infiltration has been demonstrated in patients with PitNETs [77].

Mirroring the Th1/Th2 nomenclature, macrophages are defined as polarized entities on a spectrum. At one end, there is the classically activated type, known as M1. At the other end, there is the alternatively activated type, known as M2 [78]. These two types of macrophages have different functions and exhibit distinct biomolecular features. While M1 macrophages are mainly involved in anti-tumoral processes, M2 macrophages are known for their association with tumorigenesis, invasiveness, and neovascularization [75].

Yagnik et al. demonstrated that non-functioning pituitary adenomas with cavernous sinus invasion have an M2/M1 gene expression ratio above one. Furthermore, the same authors concluded that cases with non-functioning pituitary adenomas without cavernous sinus invasion have an M2/M1 gene expression ratio below one [79]. As mentioned earlier, these findings support the statement that the M2 type is associated with tumoral invasiveness.

The polarizing stimuli of M1 are represented by interferon-gamma (IFNγ), lipopolysaccharide, tumor necrosis factor (TNF), and granulocyte–macrophage colony-stimulating factor (GM-CSF). At the same time, the primary role of these macrophages lies in activating Th1 cells, the intracellular destruction of pathogenic agents, immunological defense, immunostimulation, and tissue destruction. Regarding M1, the produced cytokines are represented by interleukins (ILs) 1, 6, 10, 12, and 23 and interleukin 1 receptor, type I; the tumoral resistance is considered high [78].

The polarizing stimuli in M2 are represented by ILs 1, 4, 10, and 13. The produced cytokines are transforming growth factor beta, TNF; interleukin 1 receptor, type II; the antagonist of interleukin 1 receptor, type I; interleukins similar to M1 but in different quantities; and other chemokines. While the tumoral resistance of M2 is poor, the identified functions of these macrophages are immunosuppression, wound healing, and remodeling of the tissues, as well as the destruction of parasitic agents followed by their encapsulation [78].

In the specific case of PitNETs, tumoral invasiveness supported by these macrophages results from TME acidification and chemokine secretion. Thus, PitNETs are characterized by lactate overproduction, leading to TME acidification. The acidification of the TME will further lead to a remodeling of tumor-associated macrophages towards an M2-like phenotype, and this chain reaction represents the beginning of tumoral invasion [80]. Thereafter, the macrophages will secrete specific chemokines that will increase the invasive feature, and in the end, after the invasive feature is enhanced, the pituitary tumor will produce even more lactate [80]. Furthermore, it has been demonstrated that in PitNETs, higher amounts of chemokines, specifically CCL17, are correlated to more significant tumoral volumes, invasiveness, and recurrence [80].

Similarly, the indirect conversion of macrophage into a specific phenotype has also been observed in functional pituitary adenomas [81]. It has been demonstrated that gonadotrophinomas drive the recruitment of tumor-associated macrophages and convert them to an M2-like phenotype. Moreover, infiltrating CD68+/CD163+ macrophages were associated with tumoral invasiveness in gonadotrophinomas, which can conclude them as an important therapeutic target in these tumors [81]. Shi et al. demonstrated that the infiltration of macrophages is associated with tumoral volumes, and PitNETs of greater volumes have a larger amount of macrophages [82]. Similarly, Liu et al. concluded that CD68+ macrophages are correlated to larger volumes and invasive features in PitNETs, while sparsely granulated somatotropinomas with more CD68+ cells have a more aggressive behavior [83].

On a cellular level, refractory pituitary lesions showed a concrete presence and resulted in the infiltration of macrophages, while in non-refractory tumors, a predominance of M2 was observed [84]. On a molecular level, in refractory tumors, an AIP mutation and an upregulation of CCL17 were demonstrated, while in non-refractory lesions, there was a normal or unidentified expression [84]. In addition, studies show that CCL5 is upregulated in *AIP*-mutation-positive PitNETs, which indirectly enhances the proliferative and invasive features of somatomammotroph cells. Thus, a biomolecular dialog between the tumor and its microenvironment is the basis of the invasive characteristic of AIP-mutation-positive lesions. At the same time, the CCL5/CCR5 pathway can be considered a potential target in the therapeutic approach [77].

A more recent study by Wu et al. brought into the spotlight the role of tumor-associated macrophages and tumor necrosis factor-alpha (TNF-α) in PitNETs with bone invasion [85]. The study concluded that a gene signature of TNF-α and tumor-associated macrophages could be a biomarker that effectively predicts bone invasion in these tumors. *C-X3-C motif chemokine receptor 1 (CX3CR1)* and *triggering receptor expressed on myeloid cells 2 (TREM2)* expression characterized the identified cells and upregulated interleukin 1B and tumor necrosis factor, aggravating bone invasion. By focusing on targeting these specific discovered entities and mechanisms, bone destruction could not only be alleviated but also avoided in the future [85].

#### 3.1.2. Lymphocytes

Various T-cell populations are found in the TME or in the draining lymphoid organs in patients with neoplastic disease, and the most experienced and capable of destroying tumoral cells is represented by cytotoxic CD8+ memory T cells. These cells are positively correlated with a good prognosis and are supported by CD4+ Th1 cells [86]. Conversely, CD4+ Th2 secretes immunosuppressive cytokines, supporting B cells or Th17 and leading to inflammation and tumoral growth and development [87]. Notwithstanding the few data available regarding immune infiltrates in PitNETs, some research detected 80% CD8+ T cells and 14% CD4+ T cells in these tumors. B lymphocytes and natural killer cells were also detected in low amounts. These results suggest a low degree of cellular immune response to PitNETs [88].

In the last decade, findings showed that the significant types of T cells are represented by regulatory T cells and cytotoxic T cells [84]. However, in the context of the TME, regulatory T cells are ubiquitous and support tumoral growth and development by blocking antitumor immune responses [2], but in the context of the TME, in PitNETs as well as in meningiomas, lower percentages or no accumulation at all have been observed, as these cells were more likely to be present in malignant tumors [89]. In cases in which the accumulation was present even in lower amounts, a significant upregulation of regulatory T cells was correlated with aggressiveness and a poor prognosis, specifically in non-functioning PitNETs [90,91].

Regarding the cytotoxic T cells, it has been stated that in addition to the cells infected by viruses, they also target neoplastic cells, inducing apoptosis and secreting perforin and granzyme. In PitNETs, their implication is still incompletely known, albeit still in research. Richardson et al. concluded that a lower amount of cytotoxic T-cell infiltration in silent type III PitNETs was correlated with recurrence and invasiveness into the neighboring anatomical structures [92]. Similarly, Iacovazzo et al. suggested that a lower amount of CD8+ lymphocytes is correlated with cavernous sinus invasiveness and treatment resistance in acromegaly patients [93]. Contrary to these findings, Wang et al. showed that higher amounts of cytotoxic T cells infiltrated in tumoral tissue were associated with invasiveness and treatment resistance in aggressive hormone-secreting PitNETs [94]. Given the different results in the medical literature, the exact role of these cellular populations and the mechanisms of action behind them are still unknown. Further research is needed to better understand whether they can impact immune diagnosis or therapeutic management in PitNETs.

#### 3.1.3. Neutrophils

Neutrophils represent up to 70% of the total circulating leukocytes, and they make up the main portion of the leukocyte infiltration in various neoplasms [95]. In neoplastic lesions, these cells follow the pattern of Th1/Th2 and M1/M2 cells, exhibiting two phenotypes described as N1, tumor-suppressive, and N2, tumor-promoting. It has been stated that the differences between these two types are related to the stage of the disease, as they become more immunosuppressive in the late stages [96]. One of the primary roles of these cells in cancers is represented by the ability to reorganize the extracellular matrix while promoting processes like neovascularization, progression, and invasiveness [97,98].

Regarding PitNETs, it has been demonstrated that the amount of neutrophils is lower compared to normal hypophysis. Furthermore, non-functioning PitNETs have more neutrophils than somatotroph adenomas due to less chemokine release and impaired chemotaxis in acromegaly [99].

Neutrophils may also be involved in tumoral aggressiveness, as clinicopathological data concluded a correlation between them, macrophages, and CD8+ T cells [99].

#### 3.1.4. Tumor-Associated Fibroblasts

Tumor-associated fibroblastic cells make up the majority of connective tissue, and their main function is represented by the remodeling of the extracellular matrix and the secretion of soluble factors such as hepatocyte growth factor, epidermal growth factor, basic fibroblast growth factor, and cytokines (interleukin 6 and stromal cell-derived factor 1) [100]. In addition, it has been demonstrated that in some types of cancer, these cells have a hormonal dependence. Furthermore, they are involved in regulating cellular motility and the metastatic spread into other organs [100].

The origin of tumor-associated fibroblasts is still under debate. While it has been concluded that multiple origins are possible, they are considered heterogeneous entities. Possible predecessors are resident tissue fibroblasts, epithelial, endothelial, and hematopoietic stem cells, as well as bone marrow-derived mesenchymal stem cells or even adipocytes [101,102].

Pituitary adenomas have multiple cell populations, such as pericytes, fibroblasts, myofibroblasts, and myoepithelial cells. Tumor-associated fibroblasts are observed in higher amounts in the stroma of refractory tumors and were also present inside the intratumoral matrix and in the capsule [103]. This is especially relevant given that the amount of cells that produce collagen is directly proportional to the grade of tumoral fibrosis. Thus, thyrotropinomas that have more cells that produce collagen have a more fibrous consistency, while somatotrophinomas and null cell adenomas that have a lower amount of collagen-producing cells have little fibrosis [103].

Despite the scarce research regarding tumor-associated fibroblasts in pituitary lesions, it is well known that the role of these cells is established not only in refractory PitNETs but also in different biomolecular behaviors. For example, the proliferation, migration, and invasion of PitNETs were suppressed by a small-interfering RNA-mediating fibroblast growth factor silencing gene [104]. Nevertheless, future studies are needed to better elucidate the entire mechanisms of action as well as the roles of tumor-associated fibroblasts in pituitary lesions [105].

#### 3.1.5. Folliculo-Stellate Cells

Accounting for up to 10% of the anterior pituitary cells, folliculo-stellate cells form networks with each other and with endothelial cells [106]. Although their complete role in PitNETs is yet to be fully discovered, it is well known that they are involved in the homeostatic regulation of the adenohypophysis, where they are associated with paracrine control in hormonal synthesis and the secretion of endocrine cells for which they provide mechanical support [107]. Various studies have also demonstrated their involvement in phagocytic activities and the secretion of cytokines and growth factors [108,109].

Furthermore, these star-shaped follicle-forming cells have scavenger activity by engulfing degenerate cells, can exhibit advanced intercellular communication abilities, and have immunoreactivity for the S100 protein [110].

In the normal hypophysis, these cells have a supporting role comparable to glial cells, and their presence in large amounts in the normal tissue neighboring the adenoma suggests their involvement in tumoral progression and cellular proliferation [111]. Moreover, a correlation between higher amounts of folliculo-stellate cell density and growth hormone levels concluded a connection between their metabolic role and hormonal secretion [111,112].

Given that they are functionally and phenotypically considered a heterogeneous cell population, research showed that a subset of the cellular population may perform professional antigen presentation and be involved in the microenvironment of tumor immunosurveillance [113].

While analyzing folliculo-stellate cells in 104 tumor samples from patients with PitNETs, Delfin et al. observed that these cells express steroidogenic factor 1 and GATA binding protein 3, which are transcription factors for gonadotrophs, and supported the theory of cellular plasticity and the transformation of hormone-producing cells to folliculo-stellate cells [114,115]. Nonetheless, future research is needed to better understand this cellular population’s exact role in PitNETs.

### 3.2. Non-Cellular Components of the TME

#### 3.2.1. Extracellular Matrix

A crucial component of the TME is represented by the extracellular matrix (ECM), a component regulated by matrix metalloproteinases and tissue inhibitors of metalloproteinases and secreted mostly by cancer-associated fibroblasts. Furthermore, it can deposit the proangiogenic factors released by proteases, such as VEGF, FGF, PDGFB, and TGFB [2].

The ECM represents approximately 60% of solid tumors and contains collagen, fibronectin, elastin, and laminin. Its major role in promoting tumoral progression and dissemination is highlighted, especially by the metalloproteinases [2]. Various matrix metalloproteinases have been associated with PitNETs, such as MMP-1 (gelatinases) and MMP-2 and MMP-9 (collagenases), MMP-3 (stromelysin), and MMP-14 (membrane type), as well as many proteins like the reversion-inducing cysteine-rich protein with Kazal motifs (RECK) and extracellular matrix metalloproteinase inducer (EMMPRIN) [22]. A disparity in matrix metalloproteinases and tissue inhibitors of metalloproteinases has been noted in invasive PitNETs, as the first ones may have an important role in tumoral growth and angiogenesis. In addition, it has been stated that the expression of IL-17 and disintegrin and metalloproteinase domain-containing protein 12 (ADAM12) is also linked to invasive PitNETs [22].

#### 3.2.2. Exosomes

Exosomes are nanovesicles that incorporate DNA, RNA, and proteins, which are embedded upon formation and transported throughout the body for intercellular communication [116]. These entities are found in the blood plasma, urine, and saliva [116]. However, a more accurate analysis has been obtained from serum exosomes, as they provide biomarkers linked to PitNETs, and it has been found that they can guide the diagnosis via the identification of the tumoral type, the prognostic, and the response to the treatment, concluding them as important biomolecular agents in the screening of PitNETs [117]. Regarding functionality within the TME, exosomes support and promote inflammatory processes, tumoral progression, neovascularization, and dissemination, while their production by the neoplastic cells is increased in hypoxic settings [2].

## 4. Translational Impact of Therapeutic Interventions

The importance of understanding how TME and its components function also has a benefit for therapeutic intervention. recent decades, various targeted agents were developed, tested, and implemented in the treatment of different cancers [118]. In PitNETs, while many of these agents are still developing or being tested, some of them have demonstrated significant benefits, and the most studied molecule in this disease is programmed death-ligand 1 (PD-L1), an immune checkpoint inhibitor [119]. Furthermore, it has been stated that PD-L1 could serve as a biomarker of response to immune checkpoint inhibitors in PitNETs [6,7].

The main recorded benefit of these targeted agents is represented by long-term disease remission without relapse after treatment discontinuation [120]. In PitNETs and pituitary carcinomas, immune checkpoint inhibitors reactivate and enhance anti-tumoral immune responses by blocking CTLA-4 or PD-1 [120]. Preclinical data demonstrated that targeting PD-L1 initiated effective antitumor immunity in aggressive ACTH-secreting pituitary tumors after reducing the plasmatic hormonal levels, decreasing tumoral volume while increasing survival rates. The results also suggested that tumor-infiltrating T cells demonstrated a pattern of checkpoint expression similar to other checkpoint blockade-susceptible tumors [121]. However, not only did preclinical data demonstrate the beneficial results of targeted agents in pituitary lesions, but they also show empirical evidence from clinical settings. Lin et al. concluded a regression of the intracranial disease by 59% and a significant plasmatic hormonal level decrease in a young female patient presenting with an aggressive ACTH-secreting pituitary tumor. The patient was initially treated with Temozolomide and capecitabine, followed by a combination of Ipilimumab and Nivolumab [8]. It is worth mentioning that the repeated attempt to introduce immune checkpoint inhibitors in the therapeutic management of functioning corticotrophinomas is backed by the evidence of higher CD8+ T infiltration in comparison to other PitNETs, which may suggest that these tumors may benefit more than other from these targeted agents [122].

Burman et al. published a study regarding 171 PitNETs and carcinomas treated using a multimodal approach, which also included Bevacizumab, immune checkpoint inhibitors, peptide receptor radionuclide therapy, the mTOR inhibitor Everolimus, and tyrosine kinase inhibitors [49]. The patients receiving immune checkpoint inhibitors as a second-line therapy achieved partial regression of pituitary lesions and metastatic lesions. However, some patients from this group achieved a transient response or disease progression. On the other hand, in patients treated with Everolimus or tyrosine kinase inhibitors, no significant results were recorded [49].

Caccese et al. also reported results of targeted agents administered in patients with different pituitary lesions [123]. Two patients with corticotroph carcinoma were given Ipilimumab and Nivolumab in combination, with partial response to treatment. Furthermore, the same study reported that two other patients with corticotrophinoma and prolactinoma were treated with Pembrolizumab monotherapy and Ipilimumab + Nivolumab. The results showed radiological and biochemical progressive diseases in both cases [123]. The possible causes of ineffective responses in the last two cases might be represented by the use of monotherapy, higher hormonal levels, or the lack of PD-L1 expression [123].

A complete response to Ipilimumab + Nivolumab of a sparsely granulated corticotrophinoma in a 57-year-old male was reported by Shah et al. in a recent article. The authors concluded a complete resolution of tumoral lesion, even 34 months postoperatively. The patient underwent a neurosurgical gross-total resection of the lesional mass, followed by proton therapy. Consequently, the neuroimaging results at the 16-month follow-up showed a tumoral recurrence, and Temozolomide administration was initiated. Despite this therapeutic management, the tumor showed significant progression, and immunotherapy with immune checkpoint inhibitors in combination was administered. Following the last step of the therapeutic approach, no residual tumor was observed [124].

Dudhamel et al. reported the case of a 60-year-old male diagnosed with prolactinoma and treated with repeated neurosurgical intervention and even daily cabergoline administration. The introduction of Temozolomide was proven ineffective, and the use of immunotherapeutic agents in combination (Ipilimumab + Nivolumab) was decided. While this was proven ineffective and stopped, the administration of Bevacizumab was decided, this time with more positive results than past evolution. The prolactin levels were stabilized, and no tumor increase was observed on neuroimaging tests, with minimal side effects [125].

Ilie et al. published an article regarding predictive factors of responses to immunotherapeutic agents, stating that the tumor type, PitNET versus pituitary carcinoma, represents the most important factor [126]. Moreover, the previous administration of Temozolomide may increase the efficacy of immune checkpoint inhibitors, given that it increases the mutational burden. Therefore, tumor mutational burden might be another predictive factor. In the same article, the authors considered that microsatellite instability and mismatch repair status could be predictive factors [126].

Wang et al. reported the case of a 41-year-old woman diagnosed with an invasive somatotronpinoma that was treated repeatedly using a neurosurgical and radiosurgical approach, with ineffective results. After initiating the treatment with Temozolomide and Apatinib, a tyrosine kinase inhibitor that selectively inhibits vascular endothelial growth factor receptor-2, the hormonal levels dropped within the normal range, while the tumoral volume decreased by 90% after one year, without any evidence of recurrence [127]. According to a report by Osterhage et al., other patients with aggressive corticotrophinomas and non-functioning PitNET also benefited from treatment with Bevacizumab after exhausting the standard therapies [128].

Bevacizumab can also play a role in familial PitNETs. It has been reported that in AIP-mutated PitNETs, a combination of Temozolomide and Bevacizumab, along with radiotherapy and pegvisomant, can result in long-term hormone secretion stabilization and can inhibit tumoral growth [129]. Furthermore, tyrosine kinase inhibitors can also play a role in familial PitNETs with MEN1 [130], and preclinical studies have demonstrated this result [131].

Potential factors that can predict treatment response to antiangiogenic agents in pituitary lesions are represented by higher vascular densities, as well as immunoreactivity of surface biomarkers (CD34 and CD31) [12].

Although clinical and preclinical studies may demonstrate a beneficial effect of targeted agents in pituitary lesions, the attempts have not gone beyond case studies, and future clinical trials are needed to draw a formal conclusion. Currently, there is one clinical study (NCT04042753) that assesses the efficacy of immune checkpoint inhibitors (Nivolumab and Ipilimumab) in patients with aggressive pituitary lesions that include PitNETs [132].

## 5. Future Perspectives Regarding Therapeutic Agents for the TME

A thorough understanding of other TME components could also greatly impact the development of new therapeutic agents. For example, in other neoplastic diseases, tumor-associated fibroblasts already represent a therapeutic target, as it is well known that they support tumor development, proliferation, and invasiveness [118]. In PitNETs, the roles of these elements are yet to be discovered. However, it has been demonstrated that they represent a source of cytokines that can impact tumor behavior. Consequently, tumor-associated fibroblasts contribute to invasiveness, aggressiveness, and angiogenesis [133]; thus, it may represent a potential future field of research.

In tumoral settings, ECM can have a major impact on therapeutic efficacy, as it can lead to treatment resistance by creating a biological barrier for therapeutic agents or by activating cellular pathways that support tumoral cells’ survival and further development [134]. Potential targets of ECM that can be considered in the future are represented by integrins and the matrix metalloproteinase system [135,136].

Secreted molecules and angiogenic factors could also represent interesting targets in PitNETs. Furthermore, somatostatin receptor ligands should be considered [12].

Besides the future opportunities to target TME components, a significant topic in the last decade has been personalized and individualized medicine in PitNETs. In addition to the already mentioned biomarkers, new potential proteomics biomarkers should be studied with the help of quantitative mass spectrometry. The study of proteomics can have multiple applications in this disease, from biomarker discovery and personalized therapeutic management to drug discovery [137,138]. However, fields beyond proteomics are also of great importance. The use of radiomics has great potential for diagnostic and prognostic purposes. Radiomics is the field that combines artificial intelligence, computer science, and radiology to amplify the accuracy of medical imaging [139,140]. In PitNETs, as in many other neoplastic diseases, the use of multi-omics should be encouraged, as the molecules from different omics levels are interconnected [137,140].

## 6. Conclusions

Understanding biomolecular mechanisms is required to provide the best therapeutic management in PitNETs. Through significant advancements, the modern era facilitates the process of diagnosis and identifying aggressive behavior in pituitary lesions. The concept of a personalized therapeutic approach is still in progress given the intricacies encountered on the road to knowledge. Nevertheless, biomolecular complexities ranging from molecular drivers to microenvironmental interactions remain an open subject that will hopefully be fully uncovered through future research.

## Figures and Tables

**Table 1:** Summary of the involved genes in PitNETs and their specific role.

GENE/PATHWAY	ROLE
*MEN1*	It has a tumor suppressor function and is involved in cellular proliferation, gene transcription, and genome stability [23].
*CDKN1B*	It is hypothesized that it is involved in tumorigenesis through unknown mechanisms and cell cycle regulation [24].
*PRKAR1A*	It is related to the tumorigenesis of somatotrophinomas, lactotrophinomas, mixed PitNETs, and corticotrophinomas, and its loss enhances protein kinase A (PKA) signaling, which is involved in transcriptional regulation, cellular progression and proliferation, and apoptosis [24].
*GPR101*	It is associated with the development of somatotroph adenomas and is highly overexpressed in pituitary lesions of X-linked acro gigantism [23,24].
*VHL*	It is involved in apoptosis and tumorigenesis and is associated with pituitary lesions in Von Hippel–Lindau syndrome [25].
*DICER1*	It promotes cellular proliferation. It is involved in tumorigenesis in DICER1 syndrome through incompletely understood mechanisms [26].
*MLH1* and *MLH2*	They are involved in tumorigenesis through incompletely understood mechanisms in Lynch syndrome [49,50].
*AIP*	It is involved in the tumorigenesis of all PitNETs through incompletely understood mechanisms [27].
*GNAS*	The only consistent mutation demonstrated in somatotropinomas is McCune–Albright syndrome [24].
*SDHx*	Germline mutations were demonstrated in hereditary pituitary lesions with pituitary adenomas, such as phaeochromocytoma/paraganglioma [28].
*PTTG1*	Its overexpression is correlated with tumor formation and progression [29,30].
*STAT3*	It supports tumorigenesis in somatotropinomas [31,32].
*CDH23*	It is involved in tumoral growth, mainly in somatotropinomas [33].
*IGSF1*	It increases the secretion of somatotropin hormone and IGF-1 levels and is associated with pituitary hyperplasia [34].
*PRDM2*	It is involved in c-Myc regulation [35].
*SLC20A*	It is associated with the activation of the Wnt–b-catenin signaling pathway [36].
*SSTR1-5* and *PR/SET Domain 2*	They are involved in the development of somatotroph adenomas. Decreased somatostatin and dopamine receptor expression levels have been associated with the tumorigenesis of somatotropinomas [37,38].
*GADD45γ*	It is involved in the development of somatotroph and nonfunctioning adenomas through DNA damage and function in the negative regulation of cell growth [38].
*RASSF1A*	It is involved in tumorigenesis through incompletely understood mechanisms [39].
*USP8*, *USP48*, and *BRAF*	Mutations are associated with dysfunctions in the adrenocorticotropic hormone and can cause the activation of the EGF signaling pathway [42].
*SFRP2*	Its overexpression reduces b-catenin and decreases Wnt signaling activity, influencing the development of corticotrophinomas [41].
*FGFR2*	It is involved in the tumorigenesis of corticotrophinomas by inducing Rb phosphorylation and the regulation of cell cycle progression via p21 and p27, primarily through deubiquitination [39,42].
*USP90*, *HDAC2*, *CABLES1*, *PTAG*, *TSP-1*, and *CASP-8*	They are involved in the tumorigenesis of corticotrophinomas through unknown mechanisms [23].
*C5orf66-AS1* and *ENC1*	Through unelucidated mechanisms, they are involved in the development of null cell adenomas [43,44].
*FAM90A1*, *ING2*, *ETS2*, *STAT6*, *MYT1L*, and *KCNK1*	They are involved in tumoral regrowth in PitNETs through unknown mechanisms [45].
*IL-6R/JAK2/STAT3/MMP9*	They are involved in cell survival, growth, proliferation, and metabolism in nonfunctioning adenomas [46].
*MEG* and *CDKN2A*	They are involved in the tumorigenesis of nonfunctioning adenomas and somatotrophinomas [23,47,48].

## References

[1] Junttila M.R., de Sauvage F.J. (2013). Influence of tumour micro-environment heterogeneity on therapeutic response. Nature.

[2] Anderson N.M., Simon M.C. (2020). The tumor microenvironment. Curr. Biol..

[3] Ayroldi E., Cannarile L., Delfino D.V., Riccardi C. (2018). A dual role for glucocorticoid-induced leucine zipper in glucocorticoid function: Tumor growth promotion or suppression?. Cell Death Dis..

[4] Yang Z., Tian X., Yao K., Yang Y., Zhang L., Liu N., Yan C., Qi X., Han S. (2023). Targeting the Tumor Immune Microenvironment Could Become a Potential Therapeutic Modality for Aggressive Pituitary Adenoma. Brain Sci..

[5] Ilie M.D., Vasiljevic A., Raverot G., Bertolino P. (2019). The Microenvironment of Pituitary Tumors—Biological and Therapeutic Implications. Cancers.

[6] Mei Y., Bi W.L., Greenwald N.F., Du Z., Agar N.Y., Kaiser U.B., Woodmansee W.W., Reardon D.A., Freeman G.J., Fecci P.E. (2016). Increased expression of programmed death ligand 1 (PD-L1) in human pituitary tumors. Oncotarget.

[7] Wang P.F., Wang T.J., Yang Y.K., Yao K., Li Z., Li Y.M., Yan C.X. (2018). The expression profile of PD-L1 and CD8(+) lymphocyte in pituitary adenomas indicating for immunotherapy. J. Neurooncol..

[8] Lin A.L., Jonsson P., Tabar V., Yang T.J., Cuaron J., Beal K., Cohen M., Postow M., Rosenblum M., Shia J. (2018). Marked Response of a Hypermutated ACTH-Secreting Pituitary Carcinoma to Ipilimumab and Nivolumab. J. Clin. Endocrinol. Metab..

[9] Voellger B., Zhang Z., Benzel J., Wang J., Lei T., Nimsky C., Bartsch J.W. (2021). Targeting Aggressive Pituitary Adenomas at the Molecular Level-A Review. J. Clin. Med..

[10] Deng L., Liang H., Burnette B., Beckett M., Darga T., Weichselbaum R.R., Fu Y.-X. (2014). Irradiation and anti–PD-L1 treatment synergistically promote antitumor immunity in mice. J. Clin. Investig..

[11] Dai C., Liang S., Sun B., Kang J. (2020). The Progress of Immunotherapy in Refractory Pituitary Adenomas and Pituitary Carcinomas. Front. Endocrinol..

[12] Ilie M.-D., De Alcubierre D., Carretti A.L., Jouanneau E., Raverot G. (2023). Therapeutic targeting of the pituitary tumor microenvironment. Pharmacol. Ther..

[13] Lamb L.S., Sim H.W., McCormack A.I. (2020). Exploring the Role of Novel Medical Therapies for Aggressive Pituitary Tumors: A Review of the Literature—“Are We There Yet?”. Cancers.

[14] Hui L., Chen Y. (2015). Tumor microenvironment: Sanctuary of the devil. Cancer Lett..

[15] Wang Z., Guo X., Gao L., Deng K., Lian W., Bao X., Feng M., Duan L., Zhu H., Xing B. (2020). The Immune Profile of Pituitary Adenomas and a Novel Immune Classification for Predicting Immunotherapy Responsiveness. J. Clin. Endocrinol. Metab..

[16] Ezzat S., Asa S.L., Couldwell W.T., Barr C.E., Dodge W.E., Vance M.L., McCutcheon I.E. (2004). The prevalence of pituitary adenomas: A systematic review. Cancer.

[17] Wang A.R., Gill J.R. (2016). The Pituitary Gland: An Infrequent but Multifaceted Contributor to Death. Acad. Forensic Pathol..

[18] Spill F., Reynolds D.S., Kamm R.D., Zaman M.H. (2016). Impact of the physical microenvironment on tumor progression and metastasis. Curr. Opin. Biotechnol..

[19] Hinshaw D.C., Shevde L.A. (2019). The Tumor Microenvironment Innately Modulates Cancer Progression. Cancer Res..

[20] Florio T. (2011). Adult pituitary stem cells: From pituitary plasticity to adenoma development. Neuroendocrinology.

[21] Dworakowska D., Grossman A.B. (2009). The pathophysiology of pituitary adenomas. Best Pract. Res. Clin. Endocrinol. Metab..

[22] Kameda-Smith M.M., Lu J. (2020). The Pituitary Tumors and Their Tumor-Specific Microenvironment. Adv. Exp. Med. Biol..

[23] Chang M., Yang C., Bao X., Wang R. (2021). Genetic and Epigenetic Causes of Pituitary Adenomas. Front. Endocrinol..

[24] Pepe S., Korbonits M., Iacovazzo D. (2019). Germline and mosaic mutations causing pituitary tumours: Genetic and molecular aspects. J. Endocrinol..

[25] Lonser R.R., Butman J.A., Kiringoda R., Song D., Oldfield E.H. (2009). Pituitary stalk hemangioblastomas in von Hippel-Lindau disease. J. Neurosurg..

[26] de Kock L., Sabbaghian N., Plourde F., Srivastava A., Weber E., Bouron-Dal Soglio D., Hamel N., Choi J.H., Park S.H., Deal C.L. (2014). Pituitary blastoma: A pathognomonic feature of germ-line DICER1 mutations. Acta Neuropathol..

[27] Marques P., Caimari F., Hernández-Ramírez L.C., Collier D., Iacovazzo D., Ronaldson A., Magid K., Lim C.T., Stals K., Ellard S. (2020). Significant Benefits of AIP Testing and Clinical Screening in Familial Isolated and Young-onset Pituitary Tumors. J. Clin. Endocrinol. Metab..

[28] Xekouki P., Pacak K., Almeida M., Wassif C.A., Rustin P., Nesterova M., de la Luz Sierra M., Matro J., Ball E., Azevedo M. (2012). Succinate dehydrogenase (SDH) D subunit (SDHD) inactivation in a growth-hormone-producing pituitary tumor: A new association for SDH?. J. Clin. Endocrinol. Metab..

[29] Gossing W., Frohme M., Radke L. (2020). Biomarkers for Liquid Biopsies of Pituitary Neuroendocrine Tumors. Biomedicines.

[30] Vlotides G., Eigler T., Melmed S. (2007). Pituitary tumor-transforming gene: Physiology and implications for tumorigenesis. Endocr. Rev..

[31] Darnell J.E., Kerr I.M., Stark G.R. (1994). Jak-STAT pathways and transcriptional activation in response to IFNs and other extracellular signaling proteins. Science.

[32] Zhou C., Jiao Y., Wang R., Ren S.G., Wawrowsky K., Melmed S. (2015). STAT3 upregulation in pituitary somatotroph adenomas induces growth hormone hypersecretion. J. Clin. Investig..

[33] Zhang Q., Peng C., Song J., Zhang Y., Chen J., Song Z., Shou X., Ma Z., Peng H., Jian X. (2017). Germline Mutations in CDH23, Encoding Cadherin-Related 23, Are Associated with Both Familial and Sporadic Pituitary Adenomas. Am. J. Hum. Genet..

[34] Faucz F.R., Horvath A.D., Azevedo M.F., Levy I., Bak B., Wang Y., Xekouki P., Szarek E., Gourgari E., Manning A.D. (2015). Is IGSF1 involved in human pituitary tumor formation?. Endocr. Relat. Cancer.

[35] Wei D., Yiyuan C., Qian L., Jianhua L., Yazhuo Z., Hua G. (2020). The absence of PRDM2 involved the tumorigenesis of somatotroph adenomas through regulating c-Myc. Gene.

[36] Li J., Dong W., Li Z., Wang H., Gao H., Zhang Y. (2019). Impact of SLC20A1 on the Wnt/β-catenin signaling pathway in somatotroph adenomas. Mol. Med. Rep..

[37] Ben-Shlomo A., Liu N.A., Melmed S. (2017). Somatostatin and dopamine receptor regulation of pituitary somatotroph adenomas. Pituitary.

[38] Srirangam Nadhamuni V., Korbonits M. (2020). Novel Insights into Pituitary Tumorigenesis: Genetic and Epigenetic Mechanisms. Endocr. Rev..

[39] Qian Z.R., Sano T., Yoshimoto K., Yamada S., Ishizuka A., Mizusawa N., Horiguchi H., Hirokawa M., Asa S.L. (2005). Inactivation of RASSF1A tumor suppressor gene by aberrant promoter hypermethylation in human pituitary adenomas. Lab. Investig..

[40] Righi A., Jin L., Zhang S., Stilling G., Scheithauer B.W., Kovacs K., Lloyd R.V. (2010). Identification and consequences of galectin-3 expression in pituitary tumors. Mol. Cell Endocrinol..

[41] Ren J., Jian F., Jiang H., Sun Y., Pan S., Gu C., Chen X., Wang W., Ning G., Bian L. (2018). Decreased expression of SFRP2 promotes development of the pituitary corticotroph adenoma by upregulating Wnt signaling. Int. J. Oncol..

[42] Chen J., Jian X., Deng S., Ma Z., Shou X., Shen Y., Zhang Q., Song Z., Li Z., Peng H. (2018). Identification of recurrent USP48 and BRAF mutations in Cushing’s disease. Nat. Commun..

[43] Yu G., Li C., Xie W., Wang Z., Gao H., Cao L., Hao L., Zhang Y. (2017). Long non-coding RNA C5orf66-AS1 is downregulated in pituitary null cell adenomas and is associated with their invasiveness. Oncol. Rep..

[44] Feng J., Hong L., Wu Y., Li C., Wan H., Li G., Sun Y., Yu S., Chittiboina P., Montgomery B. (2014). Identification of a subtype-specific ENC1 gene related to invasiveness in human pituitary null cell adenoma and oncocytomas. J. Neurooncol..

[45] Cheng S., Li C., Xie W., Miao Y., Guo J., Wang J., Zhang Y. (2020). Integrated analysis of DNA methylation and mRNA expression profiles to identify key genes involved in the regrowth of clinically non-functioning pituitary adenoma. Aging.

[46] Feng J., Yu S.Y., Li C.Z., Li Z.Y., Zhang Y.Z. (2016). Integrative proteomics and transcriptomics revealed that activation of the IL-6R/JAK2/STAT3/MMP9 signaling pathway is correlated with invasion of pituitary null cell adenomas. Mol. Cell Endocrinol..

[47] Pease M., Ling C., Mack W.J., Wang K., Zada G. (2013). The role of epigenetic modification in tumorigenesis and progression of pituitary adenomas: A systematic review of the literature. PLoS ONE.

[48] Seemann N., Kuhn D., Wrocklage C., Keyvani K., Hackl W., Buchfelder M., Fahlbusch R., Paulus W. (2001). CDKN2A/p16 inactivation is related to pituitary adenoma type and size. J. Pathol..

[49] Burman P., Trouillas J., Losa M., McCormack A., Petersenn S., Popovic V., Theodoropoulou M., Raverot G., Dekkers O.M., Guenego A. (2022). Aggressive pituitary tumours and carcinomas, characteristics and management of 171 patients. Eur. J. Endocrinol..

[50] Loughrey P.B., Baker G., Herron B., Cooke S., Iacovazzo D., Lindsay J.R., Korbonits M. (2021). Invasive ACTH-producing pituitary gland neoplasm secondary to MSH2 mutation. Cancer Genet..

[51] Iglesias P. (2024). Pituitary Apoplexy: An Updated Review. J. Clin. Med..

[52] Biagetti B., Simò R. (2022). Pituitary Apoplexy: Risk Factors and Underlying Molecular Mechanisms. Int. J. Mol. Sci..

[53] Gupta P., Dutta P. (2018). Landscape of Molecular Events in Pituitary Apoplexy. Front. Endocrinol..

[54] Safa A., Abak A., Shoorei H., Taheri M., Ghafouri-Fard S. (2020). MicroRNAs as regulators of ERK/MAPK pathway: A comprehensive review. Biomed. Pharmacother..

[55] Mou C., Han T., Zhao H., Wang S., Qu Y. (2009). Clinical features and immunohistochemical changes of pituitary apoplexy. J. Clin. Neurosci..

[56] Palmirotta R., Lovero D., Cafforio P., Felici C., Mannavola F., Pellè E., Quaresmini D., Tucci M., Silvestris F. (2018). Liquid biopsy of cancer: A multimodal diagnostic tool in clinical oncology. Ther. Adv. Med. Oncol..

[57] García-Casas A., García-Olmo D.C., García-Olmo D. (2017). Further the liquid biopsy: Gathering pieces of the puzzle of genometastasis theory. World J. Clin. Oncol..

[58] Di Ieva A., Grizzi F., Gaetani P., Goglia U., Tschabitscher M., Mortini P., Rodriguez y Baena R. (2008). Euclidean and fractal geometry of microvascular networks in normal and neoplastic pituitary tissue. Neurosurg. Rev..

[59] Ushijima T., Asada K. (2010). Aberrant DNA methylation in contrast with mutations. Cancer Sci..

[60] Chan K.C., Jiang P., Zheng Y.W., Liao G.J., Sun H., Wong J., Siu S.S., Chan W.C., Chan S.L., Chan A.T. (2013). Cancer genome scanning in plasma: Detection of tumor-associated copy number aberrations, single-nucleotide variants, and tumoral heterogeneity by massively parallel sequencing. Clin. Chem..

[61] Shapiro B., Chakrabarty M., Cohn E.M., Leon S.A. (1983). Determination of circulating DNA levels in patients with benign or malignant gastrointestinal disease. Cancer.

[62] Mouliere F., Robert B., Arnau Peyrotte E., Del Rio M., Ychou M., Molina F., Gongora C., Thierry A.R. (2011). High fragmentation characterizes tumour-derived circulating DNA. PLoS ONE.

[63] Zhang Y., Liu Y.T., Tang H., Xie W.Q., Yao H., Gu W.T., Zheng Y.Z., Shang H.B., Wang Y., Wei Y.X. (2019). Exosome-Transmitted lncRNA H19 Inhibits the Growth of Pituitary Adenoma. J. Clin. Endocrinol. Metab..

[64] Németh K., Darvasi O., Likó I., Szücs N., Czirják S., Reiniger L., Szabó B., Krokker L., Pállinger É., Igaz P. (2019). Comprehensive Analysis of Circulating miRNAs in the Plasma of Patients with Pituitary Adenomas. J. Clin. Endocrinol. Metab..

[65] Xiong Y., Tang Y., Fan F., Zeng Y., Li C., Zhou G., Hu Z., Zhang L., Liu Z. (2020). Exosomal hsa-miR-21-5p derived from growth hormone-secreting pituitary adenoma promotes abnormal bone formation in acromegaly. Transl. Res..

[66] Yu S., Wang X.S., Cao K.C., Bao X.J., Yu J. (2019). Identification of CDK6 and RHOU in Serum Exosome as Biomarkers for the Invasiveness of Non-functioning Pituitary Adenoma. Chin. Med. Sci. J..

[67] Wu Z.R., Yan L., Liu Y.T., Cao L., Guo Y.H., Zhang Y., Yao H., Cai L., Shang H.B., Rui W.W. (2018). Inhibition of mTORC1 by lncRNA H19 via disrupting 4E-BP1/Raptor interaction in pituitary tumours. Nat. Commun..

[68] Lu T., Yu C., Ni H., Liang W., Yan H., Jin W. (2018). Expression of the long non-coding RNA H19 and MALAT-1 in growth hormone-secreting pituitary adenomas and its relationship to tumor behavior. Int. J. Dev. Neurosci..

[69] Bahn J.H., Zhang Q., Li F., Chan T.M., Lin X., Kim Y., Wong D.T., Xiao X. (2015). The landscape of microRNA, Piwi-interacting RNA, and circular RNA in human saliva. Clin. Chem..

[70] Hauser B.M., Lau A., Gupta S., Bi W.L., Dunn I.F. (2019). The Epigenomics of Pituitary Adenoma. Front. Endocrinol..

[71] Ma H.S., Wang E.L., Xu W.F., Yamada S., Yoshimoto K., Qian Z.R., Shi L., Liu L.L., Li X.H. (2018). Overexpression of DNA (Cytosine-5)-Methyltransferase 1 (DNMT1) And DNA (Cytosine-5)-Methyltransferase 3A (DNMT3A) Is Associated with Aggressive Behavior and Hypermethylation of Tumor Suppressor Genes in Human Pituitary Adenomas. Med. Sci. Monit..

[72] Xue Y., Chen R., Du W., Yang F., Wei X. (2017). RIZ1 and histone methylation status in pituitary adenomas. Tumour Biol..

[73] Hua G., Yanjiao H., Qian L., Jichao W., Yazhuo Z. (2018). Detection of circulating tumor cells in patients with pituitary tumors. BMC Cancer.

[74] Allard W.J., Matera J., Miller M.C., Repollet M., Connelly M.C., Rao C., Tibbe A.G., Uhr J.W., Terstappen L.W. (2004). Tumor cells circulate in the peripheral blood of all major carcinomas but not in healthy subjects or patients with nonmalignant diseases. Clin. Cancer Res..

[75] Marques P., Grossman A.B., Korbonits M. (2020). The tumour microenvironment of pituitary neuroendocrine tumours. Front. Neuroendocrinol..

[76] Vitale I., Manic G., Coussens L.M., Kroemer G., Galluzzi L. (2019). Macrophages and Metabolism in the Tumor Microenvironment. Cell Metab..

[77] Barry S., Carlsen E., Marques P., Stiles C.E., Gadaleta E., Berney D.M., Roncaroli F., Chelala C., Solomou A., Herincs M. (2019). Tumor microenvironment defines the invasive phenotype of AIP-mutation-positive pituitary tumors. Oncogene.

[78] Allavena P., Sica A., Solinas G., Porta C., Mantovani A. (2008). The inflammatory micro-environment in tumor progression: The role of tumor-associated macrophages. Crit. Rev. Oncol. Hematol..

[79] Yagnik G., Rutowski M.J., Shah S.S., Aghi M.K. (2019). Stratifying nonfunctional pituitary adenomas into two groups distinguished by macrophage subtypes. Oncotarget.

[80] Zhang A., Xu Y., Xu H., Ren J., Meng T., Ni Y., Zhu Q., Zhang W.B., Pan Y.B., Jin J. (2021). Lactate-induced M2 polarization of tumor-associated macrophages promotes the invasion of pituitary adenoma by secreting CCL17. Theranostics.

[81] Principe M., Chanal M., Ilie M.D., Ziverec A., Vasiljevic A., Jouanneau E., Hennino A., Raverot G., Bertolino P. (2020). Immune Landscape of Pituitary Tumors Reveals Association Between Macrophages and Gonadotroph Tumor Invasion. J. Clin. Endocrinol. Metab..

[82] Shi H., Cheng Y., Ye J., Cai P., Zhang J., Li R., Yang Y., Wang Z., Zhang H., Lin C. (2015). bFGF Promotes the Migration of Human Dermal Fibroblasts under Diabetic Conditions through Reactive Oxygen Species Production via the PI3K/Akt-Rac1- JNK Pathways. Int. J. Biol. Sci..

[83] Lu J.Q., Adam B., Jack A.S., Lam A., Broad R.W., Chik C.L. (2015). Immune Cell Infiltrates in Pituitary Adenomas: More Macrophages in Larger Adenomas and More T Cells in Growth Hormone Adenomas. Endocr. Pathol..

[84] Li Y., Ren X., Gao W., Cai R., Wu J., Liu T., Chen X., Jiang D., Chen C., Cheng Q. (2024). The biological behavior and clinical outcome of pituitary adenoma are affected by the microenvironment. CNS Neurosci. Ther..

[85] Wu X., Han X., Zhu H., Li M., Gong L., Jing S., Xie W., Liu Z., Li C., Zhang Y. (2025). Single-cell transcriptomics identify a novel macrophage population associated with bone invasion in pituitary neuroendocrine tumors. J. Exp. Clin. Cancer Res..

[86] Balkwill F.R., Capasso M., Hagemann T. (2012). The tumor microenvironment at a glance. J. Cell Sci..

[87] Fridman W.H., Pagès F., Sautès-Fridman C., Galon J. (2012). The immune contexture in human tumours: Impact on clinical outcome. Nat. Rev. Cancer.

[88] Rossi M.L., Jones N.R., Esiri M.M., Havas L., al Izzi M., Coakham H.B. (1990). Mononuclear cell infiltrate and HLA-Dr expression in 28 pituitary adenomas. Tumori.

[89] Jacobs J.F., Idema A.J., Bol K.F., Nierkens S., Grauer O.M., Wesseling P., Grotenhuis J.A., Hoogerbrugge P.M., de Vries I.J., Adema G.J. (2009). Regulatory T cells and the PD-L1/PD-1 pathway mediate immune suppression in malignant human brain tumors. Neuro Oncol..

[90] Han S., Ma E., Jiang W., Lu Y., Sun X., Feng S. (2020). Overrepresentation of highly functional T regulatory cells in patients with nonfunctioning pituitary adenoma. Hum. Immunol..

[91] Huang X., Xu J., Wu Y., Sheng L., Li Y., Zha B., Sun T., Yang J., Zang S., Liu J. (2021). Alterations in CD8(+) Tregs, CD56(+) Natural Killer Cells and IL-10 Are Associated with Invasiveness of Nonfunctioning Pituitary Adenomas (NFPAs). Pathol. Oncol. Res..

[92] Richardson T.E., Shen Z.J., Kanchwala M., Xing C., Filatenkov A., Shang P., Barnett S., Abedin Z., Malter J.S., Raisanen J.M. (2017). Aggressive Behavior in Silent Subtype III Pituitary Adenomas May Depend on Suppression of Local Immune Response: A Whole Transcriptome Analysis. J. Neuropathol. Exp. Neurol..

[93] Iacovazzo D., Chiloiro S., Carlsen E., Bianchi A., Giampietro A., Tartaglione T., Bima C., Bracaccia M.E., Lugli F., Lauretti L. (2020). Tumour-infiltrating cytotoxic T lymphocytes in somatotroph pituitary neuroendocrine tumours. Endocrine.

[94] Wang P., Wang T., Yang Y., Yu C., Liu N., Yan C. (2017). Detection of programmed death ligand 1 protein and CD8+ lymphocyte infiltration in plurihormonal pituitary adenomas: A case report and review of the literatures. Medicine.

[95] Gregory A.D., Houghton A.M. (2011). Tumor-associated neutrophils: New targets for cancer therapy. Cancer Res..

[96] Fridlender Z.G., Albelda S.M. (2012). Tumor-associated neutrophils: Friend or foe?. Carcinogenesis.

[97] Demers M., Wong S.L., Martinod K., Gallant M., Cabral J.E., Wang Y., Wagner D.D. (2016). Priming of neutrophils toward NETosis promotes tumor growth. Oncoimmunology.

[98] He M., Peng A., Huang X.Z., Shi D.C., Wang J.C., Zhao Q., Lin H., Kuang D.M., Ke P.F., Lao X.M. (2016). Peritumoral stromal neutrophils are essential for c-Met-elicited metastasis in human hepatocellular carcinoma. Oncoimmunology.

[99] Marques P., Barry S., Carlsen E., Collier D., Ronaldson A., Awad S., Dorward N., Grieve J., Mendoza N., Muquit S. (2019). Chemokines modulate the tumour microenvironment in pituitary neuroendocrine tumours. Acta Neuropathol. Commun..

[100] Cirri P., Chiarugi P. (2012). Cancer-associated-fibroblasts and tumour cells: A diabolic liaison driving cancer progression. Cancer Metastasis Rev..

[101] Orimo A., Weinberg R.A. (2007). Heterogeneity of stromal fibroblasts in tumors. Cancer Biol. Ther..

[102] Sugimoto H., Mundel T.M., Kieran M.W., Kalluri R. (2006). Identification of fibroblast heterogeneity in the tumor microenvironment. Cancer Biol. Ther..

[103] Tofrizal A., Fujiwara K., Yashiro T., Yamada S. (2016). Alterations of collagen-producing cells in human pituitary adenomas. Med. Mol. Morphol..

[104] Zhou K., Fan Y.D., Duysenbi S., Wu P.F., Feng Z.H., Qian Z., Zhang T.R. (2017). siRNA-mediated silencing of bFGF gene inhibits the proliferation, migration, and invasion of human pituitary adenoma cells. Tumour Biol..

[105] Lv L., Zhang S., Hu Y., Zhou P., Gao L., Wang M., Sun Z., Chen C., Yin S., Wang X. (2018). Invasive Pituitary Adenoma-Derived Tumor-Associated Fibroblasts Promote Tumor Progression both In Vitro and In Vivo. Exp. Clin. Endocrinol. Diabetes.

[106] Allaerts W., Vankelecom H. (2005). History and perspectives of pituitary folliculo-stellate cell research. Eur. J. Endocrinol..

[107] Tapoi D.A., Popa M.L., Tanase C., Derewicz D., Gheorghișan-Gălățeanu A.A. (2023). Role of Tumor Microenvironment in Pituitary Neuroendocrine Tumors: New Approaches in Classification, Diagnosis and Therapy. Cancers.

[108] Herkenham M. (2005). Folliculo-stellate (FS) cells of the anterior pituitary mediate interactions between the endocrine and immune systems. Endocrinology.

[109] Claudius L., Yoshimi Y., Yoichiro H., Gabriel M., Koichi M. (2006). Phagocytotic removal of apoptotic endocrine cells by folliculostellate cells and its functional implications in clusterin accumulation in pituitary colloids in helmeted guinea fowl (Numida meleagris). Acta Histochem..

[110] Devnath S., Inoue K. (2008). An insight to pituitary folliculo-stellate cells. J. Neuroendocrinol..

[111] Voit D., Saeger W., Lüdecke D.K. (1999). Folliculo-stellate cells in pituitary adenomas of patients with acromegaly. Pathol. Res. Pract..

[112] Ilie M.D., Vasiljevic A., Chanal M., Gadot N., Chinezu L., Jouanneau E., Hennino A., Raverot G., Bertolino P. (2022). Intratumoural spatial distribution of S100B + folliculostellate cells is associated with proliferation and expression of FSH and ERα in gonadotroph tumours. Acta Neuropathol. Commun..

[113] Vajtai I., Kappeler A., Sahli R. (2007). Folliculo-stellate cells of “true dendritic” type are involved in the inflammatory microenvironment of tumor immunosurveillance of pituitary adenomas. Diagn. Pathol..

[114] Delfin L., Mete O., Asa S.L. (2021). Follicular cells in pituitary neuroendocrine tumors. Hum. Pathol..

[115] Wiesnagrotzki N., Bernreuther C., Saeger W., Flitsch J., Glatzel M., Hagel C. (2021). Co-expression of intermediate filaments glial fibrillary acidic protein and cytokeratin in pituitary adenoma. Pituitary.

[116] Li M., Zeringer E., Barta T., Schageman J., Cheng A., Vlassov A.V. (2014). Analysis of the RNA content of the exosomes derived from blood serum and urine and its potential as biomarkers. Philos. Trans. R. Soc. Lond. B Biol. Sci..

[117] Herrgott G.A., Asmaro K.P., Wells M., Sabedot T.S., Malta T.M., Mosella M.S., Nelson K., Scarpace L., Barnholtz-Sloan J.S., Sloan A.E. (2022). Detection of tumor-specific DNA methylation markers in the blood of patients with pituitary neuroendocrine tumors. Neuro Oncol..

[118] Bejarano L., Jordāo M.J.C., Joyce J.A. (2021). Therapeutic Targeting of the Tumor Microenvironment. Cancer Discov..

[119] Ilie M.-D., Vasiljevic A., Bertolino P., Raverot G. (2022). Biological and Therapeutic Implications of the Tumor Microenvironment in Pituitary Adenomas. Endocr. Rev..

[120] Robert C. (2020). A decade of immune-checkpoint inhibitors in cancer therapy. Nat. Commun..

[121] Kemeny H.R., Elsamadicy A.A., Farber S.H., Champion C.D., Lorrey S.J., Chongsathidkiet P., Woroniecka K.I., Cui X., Shen S.H., Rhodin K.E. (2020). Targeting PD-L1 Initiates Effective Antitumor Immunity in a Murine Model of Cushing Disease. Clin. Cancer Res..

[122] Yeung J.T., Vesely M.D., Miyagishima D.F. (2020). In silico analysis of the immunological landscape of pituitary adenomas. J. Neuro-Oncol..

[123] Caccese M., Barbot M., Ceccato F., Padovan M., Gardiman M.P., Fassan M., Denaro L., Emanuelli E., D’Avella D., Scaroni C. (2020). Rapid disease progression in patient with mismatch-repair deficiency pituitary ACTH-secreting adenoma treated with checkpoint inhibitor pembrolizumab. Anti-Cancer Drugs.

[124] Shah S., Manzoor S., Rothman Y., Hagen M., Pater L., Golnik K., Mahammedi A., Lin A.L., Bhabhra R., Forbes J.A. (2022). Complete Response of a Patient with a Mismatch Repair Deficient Aggressive Pituitary Adenoma to Immune Checkpoint Inhibitor Therapy: A Case Report. Neurosurgery.

[125] Duhamel C., Ilie M.D., Salle H., Nassouri A.S., Gaillard S., Deluche E., Assaker R., Mortier L., Cortet C., Raverot G. (2020). Immunotherapy in Corticotroph and Lactotroph Aggressive Tumors and Carcinomas: Two Case Reports and a Review of the Literature. J. Pers. Med..

[126] Ilie M.D., Villa C., Cuny T., Cortet C., Assie G., Baussart B., Cancel M., Chanson P., Decoudier B., Deluche E. (2022). Real-life efficacy and predictors of response to immunotherapy in pituitary tumors: A cohort study. Eur. J. Endocrinol..

[127] Wang Y., He Q., Meng X., Zhou S., Zhu Y., Xu J., Tao R. (2019). Apatinib (YN968D1) and Temozolomide in Recurrent Invasive Pituitary Adenoma: Case Report and Literature Review. World Neurosurg..

[128] Osterhage K., Rotermund R., Droste M., Dierlamm J., Saeger W., Petersenn S., Aberle J., Flitsch J. (2021). Bevacizumab in Aggressive Pituitary Adenomas—Experience with 3 Patients. Exp. Clin. Endocrinol. Diabetes.

[129] Dutta P., Reddy K.S., Rai A., Madugundu A.K., Solanki H.S., Bhansali A., Radotra B.D., Kumar N., Collier D., Iacovazzo D. (2019). Surgery, Octreotide, Temozolomide, Bevacizumab, Radiotherapy, and Pegvisomant Treatment of an AIP Mutation—Positive Child. J. Clin. Endocrinol. Metab..

[130] Syro L.V., Scheithauer B.W., Kovacs K., Toledo R.A., Londoño F.J., Ortiz L.D., Rotondo F., Horvath E., Uribe H. (2012). Pituitary tumors in patients with MEN1 syndrome. Clinics.

[131] Korsisaari N., Ross J., Wu X., Kowanetz M., Pal N., Hall L., Eastham-Anderson J., Forrest W.F., Van Bruggen N., Peale F.V. (2008). Blocking vascular endothelial growth factor-A inhibits the growth of pituitary adenomas and lowers serum prolactin level in a mouse model of multiple endocrine neoplasia type 1. Clin. Cancer Res..

[132] (2019). Phase II Trial of Nivolumab Plus Ipilimumab in Patients with Aggressive Pituitary Tumors.

[133] Marques P., Barry S., Carlsen E., Collier D., Ronaldson A., Awad S., Dorward N., Grieve J., Mendoza N., Muquit S. (2019). Pituitary tumour fibroblast-derived cytokines influence tumour aggressiveness. Endocr.-Relat. Cancer.

[134] Maman S., Witz I.P. (2018). A history of exploring cancer in context. Nat. Rev. Cancer.

[135] Bergonzini C., Kroese K., Zweemer A.J.M., Danen E.H.J. (2022). Targeting Integrins for Cancer Therapy—Disappointments and Opportunities. Front. Cell Dev. Biol..

[136] Yao Q., Kou L., Tu Y., Zhu L. (2018). MMP-Responsive ‘Smart’ Drug Delivery and Tumor Targeting. Trends Pharmacol. Sci..

[137] Marques-Pamies M., Gil J., Valassi E., Pons L., Carrato C., Jordà M., Puig-Domingo M. (2024). New molecular tools for precision medicine in pituitary neuroendocrine tumors. Minerva Endocrinol..

[138] Grande G., Graziani A., Toni L.D.E., Ferlin A. (2024). Proteomics for the identification of peripheral markers in pituitary disease. Minerva Endocrinol..

[139] Semenescu L.E., Tataranu L.G., Dricu A., Ciubotaru G.V., Radoi M.P., Rodriguez S.M.B., Kamel A. (2023). A Neurosurgical Perspective on Brain Metastases from Renal Cell Carcinoma: Multi-Institutional, Retrospective Analysis. Biomedicines.

[140] Aydin B., Yildirim E., Erdogan O., Arga K.Y., Yilmaz B.K., Bozkurt S.U., Bayrakli F., Turanli B. (2022). Past, Present, and Future of Therapies for Pituitary Neuroendocrine Tumors: Need for Omics and Drug Repositioning Guidance. Omics.

